# Exploring Jordanian children and parents’ awareness, behavior, and perception of pediatric oral health

**DOI:** 10.1186/s12903-023-03838-7

**Published:** 2024-01-10

**Authors:** Eman Hussein Hammouri, Asem Thabit Mustafa, Taghreed Falah Jaradat, Moa’th Mohammad Ghozlan, Mahmoud Yaseen Bani Salman, Ala’ Ahmad Ersheidat, Israa Mohammad Nawasra

**Affiliations:** 1 Department of Pediatric Dentistry, Royal Medical Services, Amman, Jordan; 2 Royal Medical Services, Amman, Jordan; 3 Department of Periodontics, Royal Medical Services, Amman, Jordan; 4 Department of Dentistry, Royal Medical Services, Amman, Jordan

**Keywords:** Oral hygiene, Pediatric oral health, Early childhood caries, Dental caries

## Abstract

**Background:**

To evaluate children’s and parents’ practice and attitude toward oral hygiene and their knowledge about oral hygiene.

**Methods:**

This cross-sectional questionnaire-based study was conducted on randomly selected children who were seen in the Pediatric dentistry clinic in different Royal Medical Services hospitals. A modified questionnaire was used to gather information from the child or parents to gather the child’s demographic data and evaluate the children’s and parents’ practice and attitude toward oral hygiene, their knowledge about oral hygiene, information about the parent and family, and oral examination, the questionnaire questions' reliability and validity were assessed by test–retest and Cronbach's Alpha test.

**Results:**

Three hundred seventy four patients were included, and the average age was 5.06 ± 3.58 SD years. Children’s and parents’ practice toward oral hygiene was inadequate where the majority (83.3%) brush their teeth occasionally, change their toothbrushes infrequently, apply toothpaste inappropriately, and less than half (47.2%) clean their tongue after teeth brushing. A significant number (73%) of candidates were aware that oral health has a significant role in their general health and can prevent dental problems. Participants agreed that maintaining a healthy mouth is an individual responsibility. The majority of participants came from large family size (the average family members 6.1 ± 1.7 SD) who live below the poverty line.

**Conclusion:**

Our study demonstrated that awareness of oral health status in children below the age of 12 was poor. Although their oral knowledge was good their attitude and behavior were inadequate. These findings urge the need for expanded, well-organized, preventive educational programs that include school’s syllabus, house visits, and hospitals for parents and children alike.

## Background

Early childhood caries (ECC) is the most common preventable chronic childhood disorder exceeding pediatric diseases in developed countries despite implemented extensive dental programs for its prevention [1–3]. Early childhood caries is a serious dental condition occurring in the first three years of life, the prevalence of which ranges from 12–70% worldwide, which is visually evident before school age [4–8]. ECC is defined as a severe type of dental caries that mostly affects children who are less than six years old. Within a short period following the eruption of primary teeth, it manifests itself by altering the smooth surfaces of those teeth. Furthermore, it is characterized by its quick progression, which leads to a severe influence on the dentition and the overall quality of life of the kid [9, 10].

According to statistics collected from all across the world, ECC continues to be very widespread, yet it is only seldom treated [11]. In the United States, the prevalence of ECC decreased significantly over the last decades among school-aged children, however, it conversely increased amongst toddlers [12, 13]. This increase among preschool children could have been induced by the wrong belief that “milk teeth caries” do not require treatment since they are eventually replaced by permanent teeth [14, 15]. In developing countries, the prevalence is even greater and exceeds the World Health Organization’s (WHO) recommended national goals [4, 16]. Although consumption of food and drinks that are high in sugar, as well as poor socioeconomic status, are important attributing factors for ECC; poor education and awareness of the necessity of pediatric oral health is considered the main factor [4, 7, 17, 18].

There seems to be an increase in the number of children in Jordan who are experiencing caries, which is a concerning development. The prevalence of caries in young infants may be significantly greater than previously thought. The frequency of caries was found to be 77.2% in children aged 5 years old, with a mean dmft of 3.9, according to a survey conducted in 2015 [19].

The prevention of ECC is much more cost-effective than alternative treatment approaches, according to research that was conducted in the past [20, 21].

Failure to identify ECC can affect a child’s quality of life (QoL) and may lead to the development of serious chronic medical illnesses later in life. For that reason, oral health awareness should commence in the perinatal period to prevent ECC via providing dental care for pregnant women, as caries is an infectious and transmittable disease postnatally. On the other side of the paradigm, early prevention can improve child growth, self-esteem, communication, and socialization [7, 22, 23].

To improve oral health and decrease the risk of ECC various community health intervention programs were implemented such as “a dental home”, the dental home should be formed no later than the age of twelve months [24, 25]. Also, referral by the primary care physician or health provider has been recommended, based on risk assessment, as early as six months of age and no later than 12 months of age [26, 27]. ‘Lift the Lip’ is another leading intervention program recommending oral health inspection during the 12-month and two-year contacts and as a component of the School Entry Health Assessment [28]. And to raise global awareness of oral health at all ages, the FDI (Fédération Dentaire Internationale) in 2007 declared the World Oral Health Day (WOHD) on the 20^th^ of March every year.

## Methods

This cross-sectional study was conducted on a research sample that was carefully chosen from the patient databases of pediatric dentistry clinics that are associated with hospitals under the Jordanian Royal Medical Services. The sample procedure used a multi-stage methodology to guarantee an accurate representation of the intended population. The first step entailed identifying possible participants from the patient databases, which contained detailed information on people who had sought pediatric dentistry care at the selected clinics. A stratified sample strategy was used to consider possible changes across various age groups. The age groups were categorized as < 6 years and 6–12 years. This ensured that the sample accurately reflected the wide range of pediatric patients. The study carefully implemented certain inclusion and exclusion criteria, which included selecting children aged 1 to 12 years and their parents who were residents of Jordan. Children with pre-existing medical issues were excluded from the study. To minimize bias and improve the generalizability of the study's results, a random selection approach was used within each stratum. The research included both children and their parents, and the selection method was designed to ensure the participation of both groups, acknowledging the significance of parental viewpoints in the field of pediatric oral health. The study made efforts to include participants from different geographical locations in Jordan, which enhanced the ecological validity of the research. The local Ethical Committee of the Royal Medical Services’ approval was granted before commencing the study. After being briefed about the research project, the parents or guardians who were responsible for the children's care signed a formal permission form. The children's verbal consent was obtained from the parents who were present throughout the course of the study.

A modified questionnaire, adapted from different researches including Al-Omiri et al. [29] Geethapriya et al. [30] Peterson et al. [31, 32] Raju et al. [33] and Stenberg et al. [34]. The questionnaire was developed using a methodical multistage process: Conducting a literature study, testing the validity, and evaluating whether to include, revise, or remove items from the questionnaire. The first version of the questionnaire was composed in English and then translated into Arabic. The translation was conducted by two proficient and independent translators. then, further translations were provided by another independent translator, and these translations were then cross-referenced with the source texts to identify and resolve any discrepancies. The questionnaire was designed specifically to ensure clarity for both parents and children. Before its implementation, the questions' reliability and validity were assessed by test–retest and Cronbach's Alpha measures. a group of 30 children participated in a pretest where they were asked to complete the questionnaire twice, with a 7-day interval between each occasion. The pretest assessed the parent’s and children's proficiency in comprehending the terminology used in the questionnaire, as well as the clarity and lack of ambiguity in the questions. The questionnaire was deemed appropriate for use due to the strong agreement in responses to the items on both occasions (Kappa test coefficient for all questions = 0.94). Some vocabulary in the questionnaire was modified somewhat before it was used in the actual survey. The survey had a total of 22 items. Parents and children were provided with a comprehensive explanation on how to evaluate their answers. In addition, the researcher was consistently accessible throughout the questionnaire's completion, and the parents and children were actively encouraged to seek his assistance anytime they needed clarification on any aspect. The questionnaire includes the following parts: part I, the child’s demographic data and includes the child’s age and gender; part II, questions related to practice toward oral hygiene by the parents/guardians or children and includes: eating habits, dental brushing, frequency of brushing per day, way of teeth brushing, amount of paste used, mouth rinse after a meal, cleaning the tongue, and the way of cleaning the tongue; part III, encompasses knowledge about oral hygiene and includes the impact of oral health on the child’s overall health, what happens if the child brushes his/her teeth irregularly, the cause of dental problems and how can they be prevented, and the role of mouth rinsing and dentist in prevention of oral problems; part IV, investigates attitude toward oral hygiene and includes individual responsibility of oral health, previous dental clinic visit(s) and the reason(s), and the importance of regular dental clinic visits for maintaining oral health; part V, composed of information about the parent/ guardian and family, which includes age, gender, job, education (school, college, or postgraduate degree), monthly income (< 500, 500–1000, or > 1000 Jordanian Dinar), number of family members; and part VI was added for oral examination to be filled by an oral hygienist under supervision of a pediatric dentist in accordance with WHO guidelines for oral health surveys (World Health Organization, 1997) and includes the followings: Standard indices of caries for deciduous and permanent teeth, DMFT (permanent teeth), dmft (primary teeth), plaque index (1–3) We used the Silness and Loe plaque index criteria to assess the dental plaque on the buccal and lingual surfaces of the six designated teeth [35], and the presence of calculus. The patients were divided into two groups according to their age, group I: < 6 years, and group II: 6- 12 year. Patients with incomplete or improperly collected data were excluded.

The data were tabulated; percentages, means, standard deviation, and frequency were analyzed using the statistical Package for Social Science 25.0 (SPSS 25.0, Inc., Chicago, IL).

## Results

A total of 374 patients were included of the surveyed 393 children, 19 were excluded because of incomplete or improper data such as mistakes in filling in the data. Most (69.3%) of the children were below the age of 6 with a slight male prevalence (54%), and the average age was 5.06 ± 3.58 SD years (1 month- 12 years) results are presented in Table 1.

**Table 1:** Demographic data of children

	**Males**	**Females**	**Total**	**Age (mean, SD)**	**Range**
< 6 Years	143(55.2)	116(44.8)	259(69.3)	3.06 ± 1.77	1 -5 years
6–12 Years	59(51.3)	56(48.7)	115(30.7)	9.85 ± 1.79	6–12 years
Total	202(54)	172(46)	374(100)	5.06 ± 3.58	1—12 years

Values mean n (%)

Descriptive results of practice, knowledge, and attitude toward oral hygiene by parents or guardians of children are presented in Tables 2, 3 and 4.

**Table 2:** Practice toward oral hygiene by parents or guardians of children

	< 6 Years	6–12 Years	Total
Eating Habits			
1 meal	0	6(5.2)	6(1.6)
2 meals	5 (1.9)	76(65.5)	81(21.7)
3 meals	254(98.1)	33(28.7)	287(76.7)
Snacks between meals	259(69.3)	115(30.7)	374(100)
Do you brush your child’s teeth?			
Yes	173(66.8)	115(100)	288(77)
No	86(33.2)	0	86(23)
How do you clean your child’s teeth?			
Toothbrush and toothpaste	171(98.8)	115(100)	286 (99.3)
Baby finger toothbrush	2(1.2)	0	2 (0.7)
How often you clean your child’s teeth?			
Once daily	19(11.0)	11(9.6)	30(10.4)
Twice daily	0	15(13.0)	15(5.2)
More than twice daily	0	3(2.6)	3(1.0)
Occasionally	154(89.0)	86(74.8)	240(83.3)
How do you brush your child’s teeth?			
Use horizontal strokes	46(26.5)	15(13)	61(21.2)
Use vertical strokes	29(16.8)	12(10.4)	41(14.2)
Both in horizontal and vertical directions	97(56.1)	86(74.8)	183(63.5)
Circular strokes	1(0.6)	2(1.7)	3(1.1)
How often you change your child’s brush?			
Once in 3 months	47(27.2)	27(23.5)	74(25.7)
Once in 6 months	54(31.2)	31(27)	85(29.5)
Once every year	20(11.6)	14(12.2)	34(11.8)
When bristles get frayed up	28(16.2)	39(33.9)	67(23.3)
What amount of paste you apply on your child’s brush?			
Full length of bristles	19(10.9)	37(32.2)	95(33)
Half-length of bristles	43(24.9)	28(24.3)	112(38.9)
Pea-sized amount	111(64.2)	50(43.5)	81(28.1)
Do you press the paste in between the bristles?			
Yes	14(8.1)	31(27)	45(15.6)
No	159(91.9)	84(73)	243(84.4)
Do you ask your child to rinse their mouth after meals?			
Yes	19(11)	10(8.6)	29(10.1)
No	149(86.1)	92((82)	241(83.6)
Sometimes	5(2.9)	13(11.2)	18(6.3)
Do you clean the tongue of your child?			
Yes	93(53.8)	43(37.4)	136(47.2)
No	80(46.2)	72(62.6)	152(52.8)
How do you clean your child’s tongue			
Tongue cleaner	0	0	0
Fingers	89(95.7)	5(11.6)	26(19.1)
Toothbrush	4(4.3)	38(88.4)	110(80.8)

Values mean n (%)

**Table 3:** Knowledge toward oral hygiene by parents or guardians of children

	< 6 Years	6–12 Years	Total
Has oral health got any role on general health of your child?			
Yes	186(71.8)	87(75.6)	273(73)
No	47(18.1)	0	47(12.6)
What does irregular tooth brushing cause?			
Decay	107(41.3)	44(38.3)	151(40.4)
Gum Disease	29(11.2)	15(13)	44(11.8)
Bad breath	36(13.9)	23(20)	59(15.7)
Stains on Teeth	15(5.8)	16(13.9)	31(8.3)
Why do your child get dental problems?			
Eating sweets and ice creams	150(57.9)	35(30.4)	185(49.5)
Not brushing properly	48(18.5)	17(14.8)	65(17.4)
Not rinsing the mouth	34(13.1)	5(4.3)	39(10.4)
Not regularly visiting a dentist	3(1.2)	2(1.7)	5(1.3)
How can we prevent dental problems?			
Avoiding sweets and sticky foods	145(56)	32(27.8)	177(47.3)
Brushing properly	73(28.2)	12(10.4)	85(22.7)
Mouth rinsing after meals	15(5.8)	4(3.5)	19(5.1)
Regularly visiting a dentist	4(1.5)	2(1.8)	6(1.6)
Do you know that cleaning the mouth can prevent tooth decay?			
Yes	254(98.1)	115(100)	369(98.7)
No	5(1.9)	0	5(1.3)
Do you know that a dentist can clean and polish your teeth?			
Yes	259(100)	115(100)	374(100)
No	0	0	0
What can be prevented by regular cleaning of your child’s mouth?			
Bleeding from gum	41(15.8)	6(5.2)	47(12.6)
Loosening of gum	14(5.4)	5(4.3)	19(5.1)
Loss of teeth	60(23.2)	7(6.1)	67(17.8)
Bad smell	51(19.7)	36(31.3)	87(23.3)

Values mean n (%)

**Table 4:** Attitude toward oral hygiene by parents or guardians of children

	< 6 Years	6–12 Years	Total
Do you think maintaining healthy mouth is an individual responsibility?			
Yes	240(92.7)	115(100)	355(94.9)
NO	19(7.3)	0	19(5.1)
Have your child visited a dentist before?			
Yes	164(63.3)	105(91.3)	269(71.9)
NO	95(36.7)	10(8.7)	105(28.1)
If yes, then for what reason?			
Decay	78/164(47.6)	44/105(41.9)	122/269(45.4)
Pain	33/164(20.1)	20/105(19)	53/269(19.7)
Filling	10/164(6.1)	16/105(15.3)	26/269(9.7)
Extraction	24/164(14.6)	13/105(12.4)	37/269(13.8)
Any other reasons	19/164(11.6)	12/105(11.4)	31/269(11.5)
Do you think it is required to visit a dentist periodically to maintain the oral health?			
Yes	158(61)	83(72.2)	241(64.4)
NO	101(39)	32(27.8)	133(35.6)

Values mean n (%)

Table 2 demonstrates the practice toward oral hygiene by the parents or guardian(s) or children. It shows that 76.7% of the children had 3 meals a day and all of them had periodic snacks, 77% brush their teeth using toothbrush and tooth paste 83.3% brush occasionally. Almost two thirds (63.5%) brush their teeth both in horizontal and vertical directions, only 25.7% change their toothbrush once every 3 months of those who brush their teeth 38.9% apply half-length of bristle tooth paste and most of them (84.4%) do not press the paste between the bristles. While 47.2% of the parents clean the tongue of their children using mainly toothbrush (80.8%), only 10.1% ask the child to rinse their mouth after meals.

Table 3 evaluated the knowledge of parents toward oral hygiene and showed that 73% know that oral health has a significant role in general health of their children. When asked what happens if your child brushes his/her teeth irregularly, 40.4% agreed that dental decay would occur and 23.8% stated that decay, gum disease, bad breath, and staining of teeth will be the end results. While half of the parents related their children's dental problems to eating sweets and ice cream and agreed that these problems can be prevented by avoiding sugars and sticky food, only 27.8% thought it was related to improper teeth brushing and mouth cleaning. Almost all candidates knew that proper oral hygiene could prevent tooth decay, bleeding and loosening of the gum, loss of teeth, and bad breath. In this part of the survey, Table 4 attitude toward oral hygiene was evaluated and it was noticed that 94.9% of candidates thought that maintaining a healthy mouth is an individual responsibility, 71.9% visited a dentist at least once before, most of them for treatment of dental decay (45.4%), and two-thirds thought it is necessary to visit a dentist periodically to maintain oral health.

Table 5 the average age of guardians was 39.3 ± 6.8 SD, 55.9% were males, and the monthly income was below 500 Jordanian Dinars in 87.4%. Housewives formed 40.4% (151/374) of the surveyed guardians, and education was at school level in 72.2%. The average family member was 6.1 ± 1.7 SD.

**Table 5:** Parent's/Guardian’s profile

**Age**	Average	39.3
	Range	23–65
**Gender of guardians**	Male	209
	Female	165
**Job**	Retired	100
	House Wife	151
	Soldier	89
	Police man/women	6
	Teacher	10
	Worker (outdoor)	18
**Education**	School	270
	College	92
	Postgraduate	12
**Income (Jordanian Dinar)**^**a**^	< 500	327
	500–1000	47
	> 1000	0
**Family Members**	Average	6.1
	Range	2- 12

^a^1 JOD = 1.41 USD

Table 6 oral examination, showed carious teeth in 240 children, 89.8% had Plaque index I, and the DMFT index for permanent teeth was for children < 6 years 0.4 and 5.17 for 6–12 years and dmft index for primary teeth was in children < 6 years 6.4 and 1.6 in 6–12 years.

**Table 6:** Oral examination

	< 6 Years		6–12 Years	Total
**Children with Missing Teeth**	131 (50.6)		53(46.1)	184 (49.2)
**Children with Carious Teeth**	192(74.1)		48(41.7)	240 (64.2)
**Children with Filled Teeth**	113(43.6)		67(58.3)	180 (48.1)
**dmft**	6.4 ± 3.8		1.6 ± 1.2	
**DMFT**	0.4 ± .03		5.17 ± 3.5	
**Plaque Index**	1.9 ± 0.3		1.31 ± .2	
**Calculus**	Yes	0	3(2.6)	3 (0.8)
	No	259(100)	112(97.4)	371 (99.2)

Values mean n (%)

## Discussion

The American Academy of Pediatric Dentistry stated that tooth-brushing should be supervised by the parents/caregivers at least until the age of 8 years. Then, there is a shift from parental guidance in oral health maintenance to personal care [36, 37]. With ECC’s increasing ubiquity among toddlers, a lot of the responsibility has fallen upon the parents or guardians to educate them about the importance of oral health. When it comes to prevention in childhood, it is not just associated with monitoring the dental health of the kid, but it also involves paying attention to the parents, who are the primary behavioral reference [38, 39].

It is of utmost importance for the school syllabus to concentrate on oral hygiene at the school level as well. Education, awareness, and learning the correct practices of oral hygiene go side by side in maintaining a child’s oral health and eventually his/her overall well-being. When the acquisition of information is followed by the application and verification of theoretical and practical abilities in terms of oral communication, school is more successful among children for content learning [40, 41]. To our knowledge, this is the first hospital-based study that comprehensively investigated the oral health knowledge, attitude, education, and practice in children and their parents over 2.5 years in Jordan, the response to the study was very good, and only 5% of the chosen sample were excluded due to missing data or inappropriate response. While previous studies conducted in Jordan involving school children showed improved dental caries and gingival diseases over the last 3 decades, the percentage of Jordanian children carrying these diseases is still higher than that of European children, this is due to greater dental knowledge, attitude, and health in European children [42–46]. The practice toward oral hygiene by parents or guardians or children showed that three quarters provide their children with 3 meals a day with snacks in between, but unfortunately, the majority (83%) brush their teeth occasionally (89.0% < 6 years Vs 74.8% 6–12 years), compared to Vishwanathaiah [47] and Al-Omari et al. [42] where a high percentage brush their teeth at least once daily. They explain this infrequent and irregular brushing by the fact that most of the children are teenagers and try to achieve independence and start building their own identities without family interference, as well as lack of both parental and child knowledge and awareness of oral health education. In our study, more than two-thirds of children were below the age of 6 years and all children who brush their teeth using a toothbrush and toothpaste; however, none of them use other important methods such as dental flossing, interdental brushes, or mouthwash and this coincides with Al-Omari et al.’s [42] conclusion who relate this to the lack of oral health education and/or the cost of hygienic products, in our study most of the parents and guardians (72.2%) had received school level education only and the majority (87.4%) have a monthly salary less than 500 Jordanian Dinars. Only 0.7% use baby finger toothbrushes, and this is also due to the lack of knowledge of such aid and its importance along with the increase in responsibilities for the parents because of the increased burdens of life (average family members 6.1). The majority (63.5%) of children brush their teeth irregularly in both horizontal and vertical directions and only 1.1% use the Fones technique, circular strokes, and the last percentage is much less than in previous studies [47]. While one quarter change their toothbrush regularly at 3-month intervals, and one quarter when bristles get frayed up, only one-third use the full-length of bristles of toothpaste, and the majority (84.4%) do not press the paste in between the bristles, these are almost similar to Vishwanathaiah’s study [48]. These findings emphasize the importance of implementing oral health education programs at schools and hospitals and even scheduling house visits to ensure that these programs are properly adopted. In our study, only 16.4% of parents ask their children, either infrequently or regularly, to rinse their mouth after each meal and this is slightly higher than previously reported studies, and almost half of the children who brush their teeth clean their tongues with their toothbrush at the end of brushing, and this is much less than in previous studies [47–49]. These findings stress the importance of mouth and tongue cleaning to improve oral health, especially before sleep.

In many countries, a significant percentage of parents and their children know about the causes and prevention of oral diseases [43, ] In our study almost three quarters of parents and their children knew that oral health has a significant role in the general health of their children, and this was less than in previous studies, this could be explained by the fact that other studies were conducted at schools and all of which had children above the age of 6 years, while in our study 18.1% of children’s parents who are less than 6 years do not know that oral health can affect the general health of the child as shown in Table 3. All parents and children agree that irregular brushing can have a significant role in oral health. Most of them (40.4%) agree that irregular brushing can cause tooth decay, but a significant percentage do not know that it may cause gum diseases, teeth staining, or bad breath, and these findings are in accordance with previous studies [29–32, 42–44, 47]. Actions are needed to increase awareness, use of other dental cleaning methods such as flossing, interdental brushes, mouth rinsing, tongue cleaning, and stop eating sticky sweets, particularly before sleep, and regular visits to the dental office. In this study, almost half of the parents and children think that dental problems are related to eating sweets and ice creams and can be prevented by avoiding sweets, sticky foods, and soft drinks. However, awareness of the importance of proper brushing, mouth rinsing, and irregular visits to the dentist were seen less frequently, 17.4%, 10.4%, and 1.3%, respectively. Interestingly, a few percentages (5.2% and 6.1%) of parents with children above 6 years knew that regular mouth cleaning can cause bleeding from gum and loss of teeth, respectively, which urges the implementation of school dental education programs.

All parents of children above 6 years agree that maintaining a healthy mouth is an individual’s responsibility, and periodic dental office visits are requisite for maintaining one’s oral health. Only 7.3% of parents whose children are below 6 years of age agree that it is the parent’s responsibility as the children are not able to properly take care of their oral hygiene, and 39% think that periodic dental office visits are not required to maintain the oral health as the teeth are replaceable. Most (91.3%) of the children above 6 years visited a dentist for treatment of dental decay (41.9%) and this reflects the poor oral hygiene of this age group as these children are teenagers and aim to achieve self-reliance and start their attempts to build their own identity without family interference [42, 47]. In comparison, more children (36.7%) below 6 years did not visit a dentist and this may be due to, as explained by previous studies, fear of dental setup, lack of toothache, lack of parental encouragement, lack of parents’ regular dental attendance which might be reflected in children’s dental attitude, parental belief and practice, lack of economic resources, lack of accessibility of dental services, and lack of both parental and child oral health education [42, 44, 47]. All these factors emphasize the vital role of the parents in properly guiding their children’s attitude toward oral health education which will have positive effects on their children's oral and general health.

Oral examination of included children revealed a slight discrepancy in missing teeth between both groups, a significant number of young children had carious teeth (< 6 years), and older age group (6–12 years) children had more filled teeth. The dmft was significant in young patients (6.4 ± 3.8) with 192 children affected by caries (74.1%) while in Al-Haddad et al. [45] 4.16 ± 3.64 and the dental decay was 87%, this may be due to the difference in age and the fact their study was concerning school children. The DMFT was 5.17 ± 3.5 in children between 6–12 years and the majority of the children had filled or missing teeth. None of the deciduous teeth has calculus and it was seen in 3 children. The plaque index for assessment of oral hygiene status was found to be similar to that of Quteish et al. [50] but different from El-Qaderi et al. [51] Rodan et al. [52], and Ballouk et al. [53].

While this study was different from previous studies in that it was carried out in a hospital and included children (males and females) before the age of 12, the sample size is representative of all Jordanian children as it includes hospitals from different locations in Jordan.

### Strengths and limitations

The research recognizes some limitations in its methodology, particularly arising from its dependence on self-reported data acquired from parents and school-aged children. The presence of differences in participants' familiarity with the questionnaire and their language proficiency creates the possibility of misinterpretation and misunderstanding of the questionnaire questions. Although the research made attempts to pretest and verify that participants understood the questionnaire, it acknowledges the difficulty of getting precise self-reported information. The investigator's constant accessibility was intended to address potential concerns, however, there is still a possibility of measuring mistakes, namely those associated with memory and misunderstanding. Moreover, there is disagreement over the representativeness of the sample of 374 children in Jordan. The study's dependence on self-reported measures, which are vulnerable to social desirability bias, carries the potential for participants to provide responses that are seen as socially acceptable rather than completely accurate. Despite these limitations, the research sought to improve dependability by doing preliminary testing and included a varied sample from several hospitals and regions in Jordan.

### Recommendations for future research

In most studies, self-reported questionnaires assessed oral health knowledge and practice, this data may not accurately reflect practice, yet it is informative. In addition to high-quality intervention and assessment studies, intervention studies are essential for evaluating the efficacy of educational programs aimed at improving oral health among students. These studies primarily concentrate on assessing outcomes such as oral health-related quality of life and dental hygiene. In addition to conventional lectures, the integration of novel techniques such as role plays, video demonstrations, and simulation models may enhance learning and provide practical instruction for oral hygiene habits. These approaches serve as effective motivating aids for children. Engaging in practical tasks such as practicing toothbrushing techniques, flossing, and using mouthwash may greatly amplify the effectiveness of initiatives aimed at promoting oral health. Frequent reinforcement of oral health knowledge is crucial for the effectiveness of educational initiatives. Introducing affordable and well-structured treatments in schools may help enhance oral hygiene and gum health, particularly in areas with little oral healthcare infrastructure also oral health programs should be compared head by head for cost-effectiveness by other treatment and prevention modalities in future studies.

## Conclusion

Our study demonstrated that the older age group (6–12 Years) has a little greater level of knowledge, consistently acknowledging the importance of oral health. Regarding behavior, children under the age of six exhibit increased parental participation in teeth brushing, often using occasional cleaning procedures. In contrast, the age group of 6–12 years demonstrates a shift towards greater independence since all parents make sure that teeth cleaning is done regularly. Both age groups have comparable perspectives on the effects resulting from inconsistent oral hygiene habits. However, the age range of 6–12 years demonstrates a slight awareness, with little variations in percentages addressing the acknowledgment of probable outcomes such as tooth decay, gum disease, halitosis, and tooth discoloration.

These results suggest extended, well-organized preventative educational programs for parents and children that incorporate school curriculum, home visits, and hospitals. We suggest creating age-appropriate courses with narrative, visual, and game elements for younger children and more in-depth explanations for older ones. To supplement learning, encourage parents to attend oral hygiene, nutrition, and preventative programs. Increase reach and engagement with mobile applications and online courses. To boost community-based efforts, host lectures or health fairs with local doctors to share success stories. Highlight the advantages of dental health education in schools for oral health and academic success. Support groups or forums may assist parents, children, and communities in learning oral health habits.

## Data Availability

The datasets used and/or analyzed during the current study are available from the corresponding author on reasonable request.

## Abbreviations

SD: Standard Deviation
ECC: Early childhood caries
WHO: World Health Organization
QoL: Quality of life
FDI: Fédération Dentaire Internationale
WOHD: World Oral Health Day
ITU: Initial Treatment Unit
DMFT (permanent teeth): Decayed (D), Missing (M), Filled (F), and Teeth (T)
dmft (primary teeth): Decayed (d), missing (m), filled (f), and teeth (t)
